# ESBL-producing *Escherichia coli* in Swedish gulls—A case of environmental pollution from humans?

**DOI:** 10.1371/journal.pone.0190380

**Published:** 2017-12-28

**Authors:** Clara Atterby, Stefan Börjesson, Sofia Ny, Josef D. Järhult, Sara Byfors, Jonas Bonnedahl

**Affiliations:** 1 Zoonosis Science Center, Department of Medical Biochemistry and Microbiology, Uppsala University, Uppsala, Sweden; 2 Department of Animal Health and Antimicrobial strategies, National Veterinary Institute (SVA), Uppsala, Sweden; 3 Public Health Agency of Sweden, Stockholm, Sweden; 4 Department of Laboratory Medicine, Karolinska Institute, Stockholm, Sweden; 5 Section of Infectious Diseases, Department of Medical Sciences, Uppsala University, Uppsala, Sweden; 6 Center for Ecology and Evolution in Microbial Model Systems, Linnaeus University, Kalmar,Sweden; 7 Department of Infectious Diseases, Kalmar County Council, Kalmar; 8 Department of Clinical and Experimental Medicine, Linköping University, Linköping, Sweden; Justus-Liebeig University Giessen, GERMANY

## Abstract

ESBL-producing bacteria are present in wildlife and the environment might serve as a resistance reservoir. Wild gulls have been described as frequent carriers of ESBL-producing *E*. *coli* strains with genotypic characteristics similar to strains found in humans. Therefore, potential dissemination of antibiotic resistance genes and bacteria between the human population and wildlife need to be further investigated. Occurrence and characterization of ESBL-producing *E*. *coli* in Swedish wild gulls were assessed and compared to isolates from humans, livestock and surface water collected in the same country and similar time-period. Occurrence of ESBL-producing *E*. *coli* in Swedish gulls is about three times higher in gulls compared to Swedish community carriers (17% versus 5%) and the genetic characteristics of the ESBL-producing *E*. *coli* population in Swedish wild gulls and Swedish human are similar. ESBL-plasmids IncF- and IncI1-type carrying ESBL-genes *bla*_CTX-M-15_ or *bla*_CTX-M-14_ were most common in isolates from both gulls and humans, but there was limited evidence of clonal transmission. Isolates from Swedish surface water harbored similar genetic characteristics, which highlights surface waters as potential dissemination routes between wildlife and the human population. Even in a low-prevalence country such as Sweden, the occurrence of ESBL producing *E*. *coli* in wild gulls and the human population appears to be connected and the occurrence of ESBL-producing *E*. *coli* in Swedish gulls is likely a case of environmental pollution.

## Introduction

*Escherichia coli* producing extended-spectrum betalactamases (ESBL) and/or carbapenemases are of great concern globally in patients and a threat to all modern healthcare. Further, community carriage of ESBL-producing *E*. *coli*, the emergence of ESBL-producing *E*. *coli* in the livestock population as well as multiple reports of ESBL-producing *E*. *coli* in wildlife show that transmission and persistence of such bacteria also occurs outside of clinical settings [1]. However, the extent to which, and how, transmission of ESBL- and carbapenemase-producing *E*. *coli* occur between the different sectors is under current investigation and debate in the scientific community [1, 2]. Several studies have reported occurrence of ESBL- and carbapenemase-producing *E*. *coli* in wild animals, primarily in wild birds. ESBL-producing *E*. *coli* in wildlife has been proposed to be a spill-over form of environmental pollution from human influenced settings and exposure to ESBL-producing *E*. *coli* from livestock through spreading of manure [1]. ESBL- and carbapenemase-producing *E*. *coli* found in wild birds often display phenotypic and genetic characteristics similar to strains in humans and thus, birds have been postulated as environmental indicators, reservoirs and possible spreaders of antibiotic resistance [3, 4]. Waterfowl and birds of prey appear to carry ESBL-producing *E*. *coli* more frequently compared to other groups of birds, possibly due to exposure to contaminated water and feed [1].

In recent years, ESBL-producing *E*. *coli* in gulls (*Laridae* spp) have been particularly studied due to their exposure to human influenced environments, migratory behavior and vast global distribution [5]. Gulls have been found to carry ESBL-producing *E*. *coli* in several studies from Europe, North America, South America and Asia and the isolates often harbor genes commonly found in strains that cause severe infections in humans [5–8]. This highlights the need to further investigate dissemination of these bacteria between gulls and human sources. Even more worrisome is that the global emergence of carbapenemase-producing *E*. *coli* is also becoming apparent in nature and such strains have recently been isolated from gulls in Spain, France and Australia [8–10].

Provided that dissemination of ESBL- or carbapenamase-producing *E*. *coli* occurs between wildlife and other sectors, the mechanism of dissemination needs to be assessed. The entire bacterium harboring resistance may be transmitted, i.e. clonal transfer. Alternatively, the genes mediating antimicrobial resistance could be transferred via plasmids or other mobile genetic elements from bacteria in one sector to bacteria more suitable to a specific host in the other sector [2].

The main objective of this study was to determine the occurrence of, and to characterize, ESBL-producing *E*. *coli* in wild gulls inhabiting urban environments in Sweden. In addition, to assess transmission we studied genetic relatedness of ESBL-producing *E*. *coli* strains found in wild gulls and compared them to strains detected in other sectors in Sweden. The current study was conducted in conjunction with a large Swedish national study reported by Börjesson *et al*. [11], investigating the dissemination of ESBL-producing *E*. *coli* in community carriers, bloodstream infection and livestock. Furthermore, a study on ESBL-producing *E*. *coli* in Swedish surface water was conducted during the same time period by Egervärn *et al*. [12]. However, there was one important difference between the current study and the other mentioned Swedish studies, (Börjesson et al. 2016, Egervärn et al. 2017) plasmid-mediated AmpC (pAmpC) betalactamase-producing *E*. *coli* were not included in the current study. The comparison of ESBL-producing *E*. *coli* isolates from wild gulls to isolates from humans, livestock and water sampled during a similar time-period and the same country is a unique feature of this study.

## Materials and methods

### Sample collection

Sample collection was performed in May-June 2013 by swirling a sterile cotton swab in freshly deposited feces from a variety of Gulls *(Larus marinus*, *Larus argentatus*, *Larus canus and Croicocephalus ridibundus)* (n = 96) in Malmö (Lat 55.6° Long 13.0°) and Black-headed Gulls *(Croicocephalus ridibundus)* (n = 74) in Gothenburg (Lat 57.7° Long 11.9°). Swabs were placed in cryovials containing Luria Berthani broth and glycerol (20%) and stored frozen at -80°C until further analyses was performed. The sampling did not require a field permit.

### Isolation and verification of ESBL-producing *Escherichia coli*

Fecal samples were enriched in Brain Heart Infusion broth with 16 mg/L Vancomycin and subsequently cultivated on chromID^R^ ESBL bacterial plates (Biomerieux). Species identity of presumptive *E*. *coli* was assessed using MALDI-TOF (Biotyper, Bruker Corporation, The Netherlands). To confirm ESBL phenotype, isolates were prepared and spread on Muller-Hinton agar (Linnaeus University in Kalmar) according to EUCAST disc diffusion method for antimicrobial susceptibility testing and five antibiotic discs—amoxicillin/clavulanic acid 30/1 μg, cefotaxime 5 μg, ceftazidime 10 μg, cefepime 30 μg, and cefoxitin 30 μg–were placed on the plate. Specific inhibition of bacterial growth around the antibiotic discs was used to identify ESBL phenotypes [13, 14].

### Characterization of ESBL-producing *Escherichia coli*

ESBL-genes were identified using the Check-MDR microarray system (CT-101 or -103) (Checkpoint BV, Wageningen, the Netherlands) and sequenced using Sanger sequencing [15]. All isolates were subjected to multilocus sequence typing (MLST) according to protocols at the University of Warwick web site (http://mlst.warwick.ac.uk/mlst/dbs/Ecoli). Alleles and STs were determined using BioNumerics 7.0 MLST Plug-in (Applied Maths, Gent, Belgium).

All isolates were tested for susceptibility to additional 13 antibiotics by disc diffusion according to EUCAST disc diffusion method for antimicrobial susceptibility testing and/or using VetMIC GN-mo panels according to the manufacturer (SVA, Uppsala) (Table 1), with the exception of fosfomycin were susceptibility was determined by Etest (bioMérieux, Sweden). Epidemiological cut-off values for resistance according to EUCAST (www.eucast.org) were used. Isolates with decreased susceptibility to ≥ 3 antibiotic classes in including beta-lactam antibiotics were classified as multiresistant.

**Table 1:** Characteristics of ESBL-producing *Escherichia coli* in wild gulls habituating in Swedish urban environments in 2012. Susceptibility to ampicillin (Am), ciprofloxacin (Ci), nalidixic acid (Nal), gentamycin (Gm), streptomycin (Sm), tetracycline (Tc), flophenicol (Ff), colistin (Cs), sulfamethoxazole (Su), trimethoprime (Tm), chloramphenicol (Cm), kanamycin (Km), cefotaxim (Ctx) and ceftazidime (Caz), and was determined by microdilution. Susceptibility to, cefoxitin (Fox), tobramycin (Nn), piperacillin/Tazobactam (Tzp), amoxicillin/Clavulanic acid (AmC), tigecycline (Tgc), nitrofurantoin, meropenem, amikacin, Ertapenem and imipenem was determined by disc diffusion. Susceptibility to fosfomycin was determined by E-test. Epidemiological cut-off values for resistance according to EUCAST (www.eucast.org). All isolates were resistant to ampicillin and cefotaxime.

City	β-lactamase gene	MLST type	Antibiotic resistance	Replicon type	Resistance
				(pMLST of IncI1)	transformants ^e^
Malmö	*bla*_CTX-M-32_	ST681	AmC, Caz	^a^	
Malmö	*bla*_CTX-M-27_		Tc, Sm, Su, Tm	IncFIB/FII	Tc, Su, Su, Tm
Malmö	*bla*_CTX-M15_	ST10	Ci, Tc	^a^	
Malmö	*bla*_SHV-12_	ST10	Ci, Tm, Nal, Tc	IncI1 (pST3)	-
Malmö	*bla*_CTX-M-15_	ST10	Ci, Tc, Caz	^a^	
Malmö	*bla*_CTX-M-15_	ST3268	Ci, Sm, Su, AmC, Caz	^b^	Ci, Su, Tm
Malmö	*bla*_CTX-M-15_	ST540	Ci, Sm, Su, Tm, Tc, AmC, Caz	^b^	-
Malmö	*bla*_CTX-M-15_	ST93	Ci, Tm, Nal, Tc, Caz	IncFIA, IncFIB	Tc, Tm
Malmö	*bla*_CTX-M-32_	ST681	AmC, Caz	^a^	
Malmö	*bla*_CTX-M-55_	ST58	Nal, Tc, Gm, Nn, Ci, AmC, Caz	IncFIA/FIB/FII	Ci, Gm, Tc
Malmö	*bla*_CTX-M-55_	ST58	Ci, Nal, Tc, Gm, AmC, Caz	IncFIA/FIB/FII	Ci, Gm, Tc
Malmö	*bla*_CTX-M-1_	ST10	-	^a^	
Malmö	*bla*_CTX-M-1_	ST10	Nal, Tc, Gm, Caz	^a^	
Gothenburg	*bla*_CTX-M-15_	ST617	Ci, Su, Tm, Nal, AmC, Caz	^a^	
Gothenburg	*bla*_CTX-M-55_	ST155	Ci, Ff, Sm, Su, Km, Tm, Nal, Tc, Cm, Gm, Nn, AmC, Caz	IncFIB/FII	Gm, Tc, Su, Tm
Gothenburg	*bla*_CTX-M-14_		Ci, Ff, Su, Tgc, Tc, Cm, Gm, AmC	IncHI1	Ci, Ff, Gm, Tc, Su, Cm
Gothenburg	*bla*_CTX-M-15_	ST767	Ci, Sm, Su, Km, Tm, Nal, Tc, Cm, Gm, AmC, Caz	IncI1 (pST175)	-
Gothenburg	*bla*_SHV-12_	ST540	Ci, Sm, Su, Tm, Tc, AmC	^b^	Ci
Gothenburg	*bla*_CTX-M-15_	ST155	Ci, Caz	IncK	Ci
Gothenburg	*bla*_SHV-12_		Ci, Su, Cm, AmC	^b^	Ci, Su, Cm
Gothenburg	*bla*_CTX-M-15_	ST10	Ci, Km, Nal, Gm, Nn, Fox, Caz, Tzp, AmC	^b^	-
Gothenburg	*bla*_CTX-M-14_	ST10	Ci, Su, Tm, Nal, Tc, AmC	IncBO	-
Gothenburg	*bla*_CTX-M-15_	ST131	Ci, Sm, Su, Km, Tm, Nal, Tc, Gm, Nn, AmC, Caz	^a^	
Gothenburg	*bla*_CTX-M-15_	ST38	Ci, Sm, Su, Tm, Nal, AmC, Caz	^a^	
Gothenburg	*bla*_CTX-M-14_	ST38	Ci, Sm, Su, Tm, Nal, AmC	^a^	
Gothenburg	*bla*_CTX-M-15_		Tm, Nal, Tc, Cm, Gm, Nn, Tzp, AmC, Caz ^d^	^c^	
Gothenburg	*bla*_CTX-M-15_	ST636	Tm, Nal, Tc, AmC, Caz ^d^	^c^	
Gothenburg	*bla*_CTX-M-14_	ST58	Nal, AmC, Caz ^d^	^c^	
Gothenburg	*bla*_CTX-M-14_	ST155	Tc, Cm, Gm, AmC ^d^	^c^	

^a^Non-transferable with transformation.

^b^Non-typable using PCR-based plasmid replicon typing.

^c^Transformation and replicon typing was not performed.

^d^Microdilution was not performed.

^e^The recipient cell ElektroMax ™ DH108 ™ (Gibco Invitrogen) naturally resistant to streptomycin.

### Transformation and characterization of plasmids carrying ESBL-producing *E*. *coli*-genes

On a subset of randomly selected isolates, transfer of plasmids carrying ESBL-producing *E*. *coli*-genes was assessed by electroporation to ElectroMax DH10B cells (Life Technologies, Carlsbad, CA, USA) and transformation was confirmed by detection of the same genes as previously described [16]. All transformants positive for an ESBL gene were tested for plasmid replicon types using the Diatheva PBRT-kit (Fano, Italy). For transformants positive for incompatibility group incI1, the plasmid was subjected to plasmid MLST (pMLST) according to the Plasmid MLST database (http://pubmlst.org/plasmid/).

Transformants and corresponding donor cells were also tested for susceptibility to 14 antibiotics by microdilution according to CLSI standard [16] using VetMIC GN-mo panels (SVA, Uppsala) (Table 1).

## Results

### Occurrence of ESBL-producing *E*. *coli* in wild gulls

ESBL-producing *E*. *coli* were detected in 13 out of 96 sample collected from Malmö and 16 out of 74 samples collected from Gothenburg, yielding occurrence frequencies of 14% and 22% respectively and 17% combined. None of the 29 isolates were resistant to carbapenems.

### Characteristics of ESBL-producing *E*. *coli* in wild gulls

All 29 ESBL-producing *E*. *coli* isolates were characterized regarding MLST-type, ESBL-genotype and susceptibility to antibiotics (Table 1). ESBL-producing *E*. *coli*-isolates belonged to 13 different MLST-types: ST10 (n = 7), ST58 (n = 3), ST155 (n = 3), ST38 (n = 2), ST540 (n = 2), ST131 (n = 1), ST681 (n = 1), ST3268 (n = 1), ST93 (n = 1), ST681 (n = 1), ST617 (n = 1), ST676 (n = 1), ST636 (n = 1) and non-typable (n = 4). The genotypic characterization revealed seven different genes encoding for ESBLs; *bla*_CTX-M-15_ (n = 13), *bla*_CTX-M-14_ (n = 5), *bla*_CTX-M-55_ (n = 3), *bla*_SHV-12_ (n = 3), *bla*_CTX-M-1_ (n = 2), *bla*_CTX-M-32_ (n = 2) and *bla*_CTX-M-27_ (n = 1). The isolate of MLST-type ST131 carried the *bla*_CTX-M-15_ gene.

Out of the 29 isolates, 25 isolates were subjected to gene transfer and subsequently replicon typing. Plasmid transfer and replicon typing were achieved in 10 isolates (40%) (Table 1). The non-transferrable isolates harboured *bla*_CTX-M-15_ (n = 5), *bla*_CTX-M-1_ (n = 2), *bla*_CTX-M-32_ (n = 2) and *bla*_CTX-M-14,_ (n = 1). Isolates resulting in unsuccessful plasmid replicon typing harboured *bla*_CTX-M-15_ (n = 3) and *bla*_SHV-12_ (n = 2). Two genes, one *bla*_CTX-M-15_ and one *bla*_SHV-12_, were identified on an IncI1 plasmid, these two were further characterized regarding plasmid-MLST and belonged to pST175 and pST3 respectively.

Susceptibility to 10 antibiotic classes was assessed and 83% of ESBL-producing *E*. *coli*-isolates were classified as multiresistant, i.e. resistant to ≥ 3 classes of antibiotics. Resistance to fluoroquinolones, aminoglycosides, tetracycline, trimethoprim and sulfa-methoxazole were common characteristics. All isolates were susceptible to amikacin, colistin, fosfomycin, nitrofurantoin, ertapenem, meropenem and imipenem.

### Comparison to other sectors

The characteristics of the isolates from the current study (Table 1) were compared to isolates from two other studies conducted in the same year in Sweden; a large Swedish national study reported by Börjesson *et al* [11], investigating the dissemination of ESBL-producing in community carriers, bloodstream infection and livestock as well as a study by Egervärn *et al* [12] investigating ESBL-producing *E*. *coli* in Swedish surface water. Comparisons were made at the level of ESBL gene, plasmid replicon type, MLST and the combinations of all mentioned.

#### ESBL-producing E. coli genes

**Fig 1:**
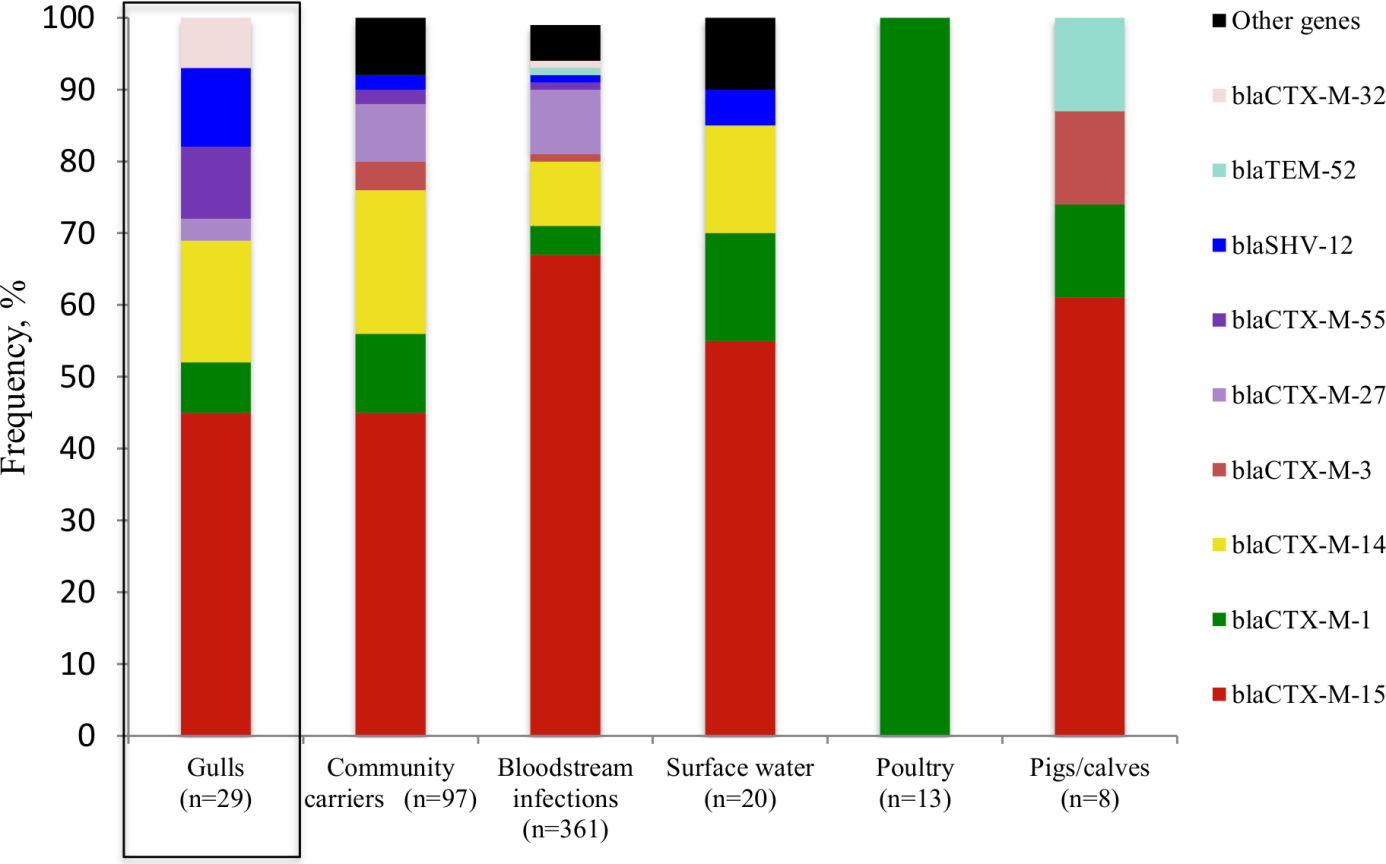
Frequency of overlapping ESBL genes in Swedish gulls (current study), community carriers, bloodstream infections, poultry, pigs/calves (Börjesson *et al*. [11]) and surface waters (Egervärn *et al*. [12]).

In Fig 1, the frequency of ESBL-producing *E*. *coli* genes in different sectors is visualized. The vast majority, 89%, of identified genes in gulls were of CTX-M-type and 11% were of SHV-type. The dominating gene in Swedish gulls, *bla*_CTX-M-15_, was detected in 45% of the isolates and *bla*_CTX-M-14_ was the second most common gene, detected in 17% of isolates. *bla*_CTX-M-15_ and *bla*_CTX-M-14_ were the most prevalent genes isolated from community carriers and bloodstream infections [11] and *bla*_CTX-M-15_ was the most common gene in Swedish surface water [12]. ESBL-producing *E*. *coli* isolates from poultry were exclusively of *bla*_CTX-M-1_ type and isolates from pigs/calves carried genes similar to the human and environmental sectors.

#### Plasmids and combinations of plasmids and genes

**Fig 2:**
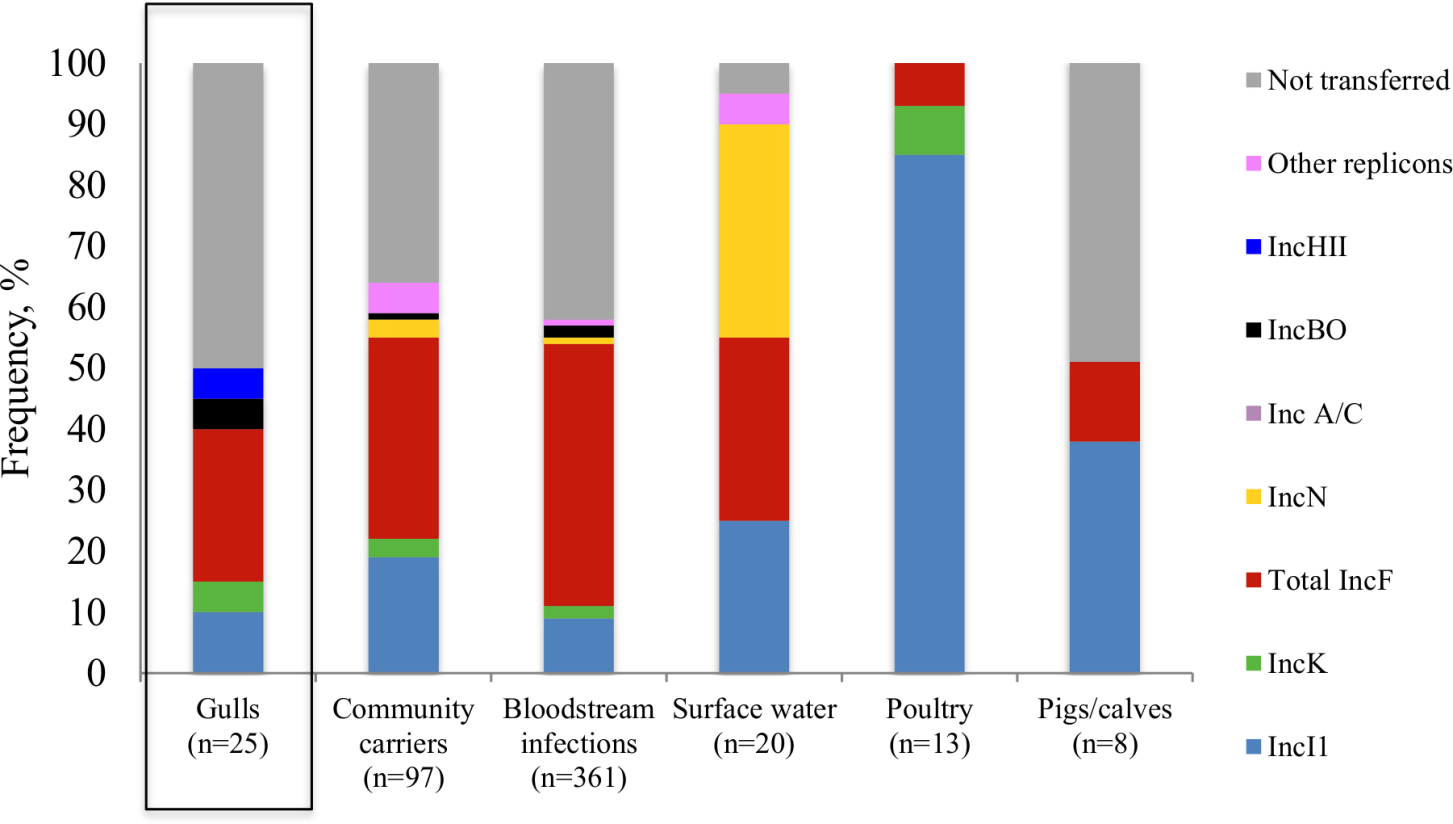
Frequency of overlapping plasmid replicon types containing ESBL genes in Swedish gulls (current study), community carriers, bloodstream infections, poultry, pigs/calves (Börjesson *et al*. [11]) and surface waters (Egervärn *et al*. [12]).

In Fig 2, the frequency of plasmid replicon type connected with plasmid carrying ESBL-producing *E*. *coli* genes in different sectors is visualized. ESBL genes identified in *E*. *coli* isolates from gulls were mainly connected to plasmids belonging to different IncF replicon types and IncI1. The distribution of plasmid types in gulls was similar to the distribution of plasmid types in community carriers and bloodstream infections. A high percentage of plasmids from gulls (50%), community carriers (36%), bloodstream infections (42%) and pigs/calves (50%) could not be transferred.

Eight isolates from gulls were successfully assigned a replicon type and genotype, out of which six isolates displayed combinations of plasmid replicon types and genes that were found in healthy human carriers and/or bloodstream infection in the same year; IncI1-*bla*_CTX-M-15,_ IncFIA/B-*bla*_CTX-M-15,_ incK-*bla*_CTX-M-15_, incB/O-*bla*_CTX-M-14,_ IncBO-*bla*_CTX-M-14_ and incFIB/II-*bla*_CTX-M-55_ (Table 2). Three isolates from gulls had identical plasmid/gene combinations to isolates found in Swedish surface water. Only one isolate from pigs/calves had a combination of plasmid/gene that was identical to one isolate found in gulls, IncI1-*bla*_CTX-M-15_. Isolates from poultry displayed no plasmid/gene overlap when compared to isolates from gulls. To further characterize a selected proportion of isolates, plasmids of IncI1 type (n = 2) were subjected to pMLST. IncI1-pST3 carried the *bla*_SHV-12_ gene and IncI1-pST175 carried the *bla*_CTX-M-15_ gene. These two combinations of pMLST, plasmid replicon type and gene were not found among isolates from other sectors.

**Table 2:** Number (%) of overlap plasmid-replicon type /gene combination in ESBL-producing *E*. *coli* isolates from Swedish gulls (current study), community carriers, bloodstream infections, poultry, pigs/calves (Börjesson *et al*. [11]) and surface water (Egervärn *et al*. [12]).

Plasmid	Gene variant in plasmid	Swedish gulls (n = 25)	Community carriers (n = 97)	Bloodstream infections (n = 361)	Swedish surface water (n = 27)	Swedish calf/pig (n = 8)
**IncI1**	*bla*_CTX-M-15_	1 (4)	8 (8)	13 (3)	2 (7)	1 (9)
	*bla*_SHV-12_	1 (4)	-	-	2 (7)	-
**IncFIA/B**	*bla*_CTX-M-15_	1 (4)	6 (6)	44 (11)	1 (4)	-
**IncK**	*bla*_CTX-M-15_	1 (4)	1 (1)	2 (0.5)	-	-
**IncBO**	*bla*_CTX-M-14_	1 (4)	-	5 (1)	-	-
**IncFIB/II**	*bla*_CTX-M-27_	1 (4)	8 (8)	31 (8)	-	-
**IncFIB/II**	*bla*_CTX-M-55_	1 (4)	-	3 (1)	-	-

#### ST-types and combinations of ST-types, plasmids and genes

**Fig 3:**
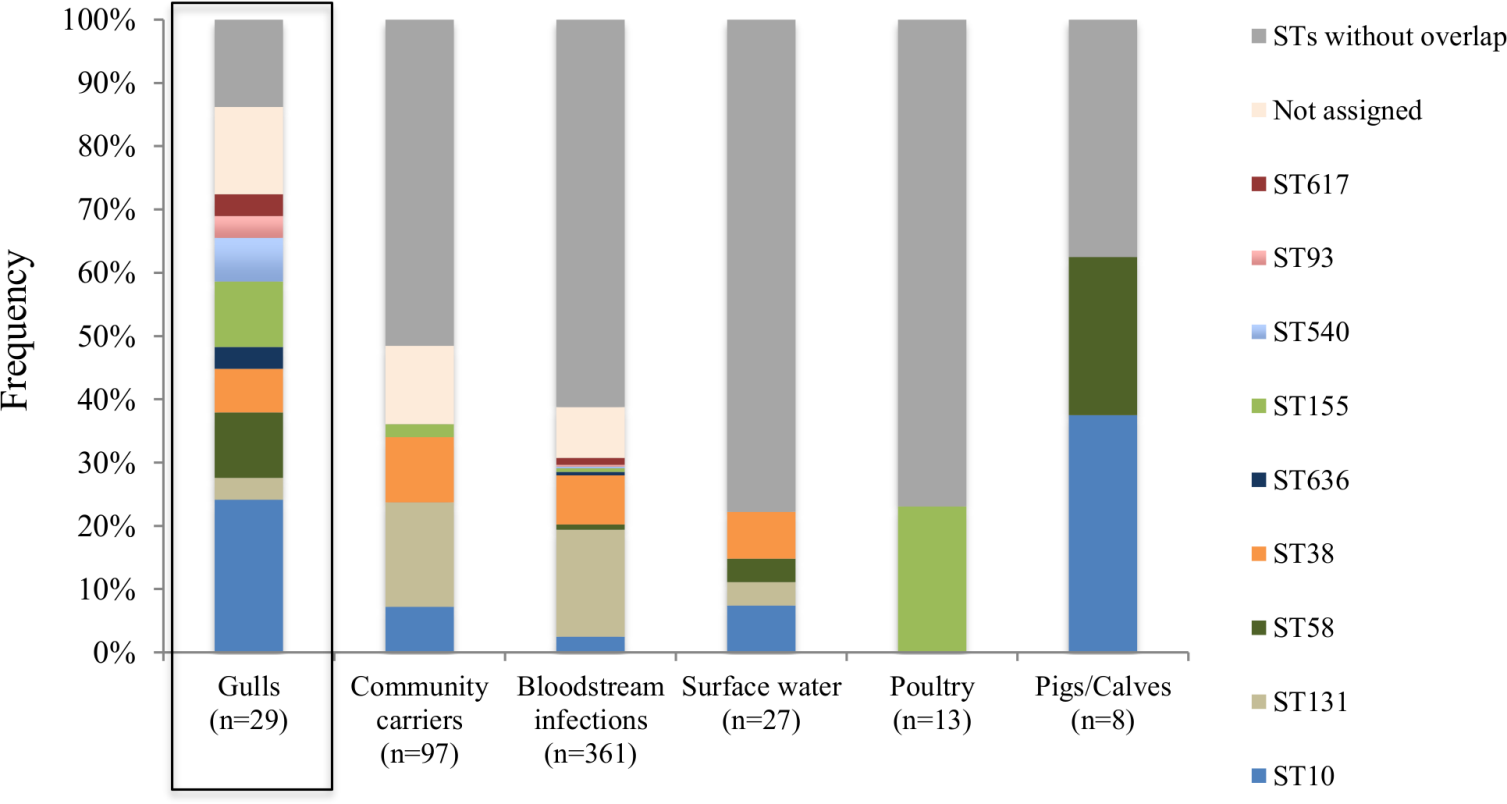
Frequency of multi-locus sequence types (MLST) in ESBL producing E. coli isolates from Swedish gulls (current study), community carriers, bloodstream infections, poultry, pigs/calves (Börjesson *et al*. [11]) and surface waters (Egervärn *et al*. [12]).

Nine out of twelve ST-types identified from gulls were detected in Swedish community carriers and/or bloodstream infections in the same year (Fig 3). Four out of the overlapping nine ST-types were also found in Swedish surface water in the same year [12]. ST10 and ST58 were found in Swedish calves and ST155 in Swedish poultry sampled in the same year [11]. The combination ST131-*bla*_CTX-M-15_ was found in one gull in this study. This combination was very common in bloodstream infections where ST131-*bla*_CTX-M-15_ was found in 31% of all isolates. When comparing clonal distributions, defined as identical ST, plasmid, and gene, of *E*. *coli* isolates from the different sectors, no overlapping combinations could be identified.

## Discussion

In this study, we found that the genetic characteristics of ESBL-producing *E*. *coli* in Swedish wild gulls mirrors those found in humans living in Sweden. The two most common ESBL genes identified in *E*. *coli* isolates from Swedish gulls were *bla*_CTX-M-15_ and *bla*_CTX-M-14_, which are the same genes most frequently observed in isolates from community carriers and bloodstream infections in Sweden. Furthermore, isolates from Swedish gulls and humans carried ESBL-plasmids belonging to IncF- and IncI1-types, and in both populations, a large proportion of genes belonging to *bla*_CTX-M-15_ could not be transferred. The unsuccessful transfer of *bla*_CTX-M-15_ genes could be due either to a non-transferrable plasmid, or that the gene was chromosomally located, the latter have previously been described to be relatively common in ESBL-producing *E*. *coli* harbouring *bla*_CTX-M-15_ [17]. The genes *bla*_CTX-M-15_*/bla*_CTX-M-14_ and plasmids IncF/IncI1 were also common in isolates from surface water and healthy pigs/calves, previously recognized as groups carrying ESBL-producing *E*. *coli* strains with characteristics similar to strains carried by humans [11, 12]. The genetic characteristics of ESBL-producing *E*. *coli* in gulls and humans were generally different to the characteristics of poultry isolates. In Swedish poultry, isolated ESBL-producing *E*. *coli* carried *bla*_CTX-M-1_ genes. However, the clear majority of cephalosporin resistant *E*. *coli* in Swedish poultry are of pAmpC-type and harbour *bla*_CMY-2_ genes [18]. No *bla*_CMY-2_ genes were detected in Swedish gulls in this study, implying that genetic transfer between the wild bird population and the poultry population is limited. It should nevertheless be noted that the current study used chromID ESBL agar plates, which are primarily selective for ESBL-producing isolates. It is therefore likely that we underestimated the occurrence of *bla*_CMY-2_ and other pAmpCs. Though if strains carrying *bla*_CMY-2_ had been common in Swedish gulls it is highly likely that at least a fraction would have been detected using our methodology. In concordance with our findings, most previous studies have also reported a dominance of *bla*_CTX-M-type_ genes in gulls [6, 19–21], but *bla*_CMY-2_ has been identified from gulls in Spain and Florida [22, 23]. Our study shows that Swedish gulls carry isolates showing genetic similarities to isolates from humans in Sweden; this indicates that the migratory Swedish gull population is domestically infected. However, the genetic characteristics of the bacteria from Swedish gulls and humans are also similar to those found in other European countries, prompting a cautious interpretation. For example, the most common ESBL-genes in Swedish gulls and humans, *bla*_CTX-M-15_ and *bla*_CTX-M-14,_ are also common in human isolates from Germany [24, 25], France [26], Switzerland [27] and the Netherlands [28]. While in European farm animals, *bla*_CTX-M-1_ appears to be the most common ESBL gene in cattle, pigs and poultry, and in poultry the pAmpC gene *bla*_CMY-2_ also appears to be widespread [29].

There was a high heterogeneity of STs found in gulls in this study. All of the STs have previously been detected in isolates from humans (MLST Database of Warwick, 2017-04-01), and a majority of them were detected in isolates from Swedish humans in the same year [30]. The similarities in genetic characteristics in isolates from gulls and humans indicate transmission of ESBL-producing *E*. *coli* between the two groups. However, despite the overlapping sequence types, ESBL-genes and ESBL-carrying plasmids in gulls and humans, there was no evidence of clonal spread due to the absence of identical combinations of sequence type/gene/plasmid. Although one should be aware that the lack of direct overlap between the different sector could also be due to the small sample size in the current study. The fact that only one isolate was collected per sample in each of the studies, likely also influenced the outcome. However, the absent evidence of clonal spread and heterogeneity of ST-types could also suggest dissemination of genes encoding ESBLs rather than clonal transmission of resistant bacteria. ESBL-genes are transferred easily between bacteria through mobile genetic elements, primarily via horizontal transfer of plasmids [31]. A high diversity of ESBL-producing *E*. *coli* in communities is a common finding in previous studies, especially pronounced in CTX-M-type ESBL-producing *E*. *coli* [32]. On the contrary, two findings from our study could be consistent with clonal transfer–firstly, one of the gull isolates from Gothenburg might be derived from the successful sub-clone of *bla*_CTX-M-15_ -producing *Escherichia coli* ST131. This sub-clone has spread as a pandemic among humans, often identified as the pathogen in severe bloodstream infections [33]. Secondly, ST617, ST38 and ST10, found in one, two and seven gulls respectively, are also sequence types with clinical importance in human medicine [34–37]. A recent study also described high relatedness of clinical isolates of ST38 from UK and one isolate of ST38 isolated from a Mongolian wild bird [36]. In that study it was also shown that the ST38 isolates carried the genes *bla*_CTX-M-14_ and *bla*_CTX-M-15_ chromosomally, and in the current study these same two genes in ST38 were non-transferable with transformation (Table 1).

In our study, ESBL-producing *E*. *coli* occurred in 14% and 22% of gulls sampled in the Swedish cities Malmö and Gothenburg, respectively, 17% combined. This is slightly higher than the reported occurrence in gulls sampled in Stockholm in 2010, 9% [38], but at a similar level as gulls sampled in the Swedish city Hudiksvall in 2009, 21% [5]. Thus, it appears the occurrence frequency in Swedish gulls is relatively stable the last few years, although one should be careful in making direct comparisons due to small sample sizes (200–300) and different sampling locations and to some extent species of gulls. Interestingly enough the occurrence of ESBL-producing *E*. *coli* in wild gulls was therefore more than three times higher than that described in Swedish human carriers [30]. This finding is also in concordance with studies performed in other European countries, but with the difference that the ratio of ESBL-producing *E*. *coli* in gulls versus humans is often even higher [5]. This difference in carriages might be due to a higher degree of exposure and possible accumulation of resistant bacteria of the gulls; e.g. proximity to human activity has been identified as a risk factor for occurrence of antibiotic resistant bacteria in wild animals in several studies [39–42]. One such human activity could be wastewater treatment plants (WWTPs) which have been recognized as hotspots for the accumulation, selection, and spread of antibiotic resistance to the environment [43]. This as gulls are often observed in connection to open WWTP basins containing untreated faecal material from entire cities. After sewage treatment in WWTPs, bacterial load is reduced by approximately 99%, but ESBL-producing *E*. *coli* are still frequently detected in rivers downstream of WWTPs and exemplifies a direct influx of antibiotic resistance from humans to the environment [44]. In this study, the isolates from gulls and humans showed high similarities to those originating from Swedish surface waters, suggesting WWTPs as one possible source. Comparable a recent Norwegian study described that ST131, ST10 and ST38 carrying the genes *bla*_CTX-M-1_, *bla*_CTX-M-14_ and *bla*_CTX-M-15_ were frequently identified from surface waters, wastewaters and human UTIs [37]. The same gene and MLST combinations were also frequent in Swedish gulls, humans and surface waters (Table 1, Fig 1, Fig 3).

There is evidence supporting a human-to-wild bird directed transmission of antibiotic resistant bacteria/antibiotic resistant genetic elements, but an important question is whether birds can also transmit antibiotic resistance to the human population? As pointed out by Guenther *et al* [1], in contrast to the human population, there is no sewage system for bird feces and droppings are shed directly into the environment, potentially exposing human and animal populations to resistant bacteria. Interestingly, wild birds have been found to transmit *Campylobacter* to crops of peas which led to an outbreak of Campylobacteriosis in humans in Alaska [45] and *Campylobacter* from wild birds have been isolated from children´s playground in New Zealand [46]. Furthermore, a case of bovine salmonellosis in a Japanese dairy farm was associated with *Salmonella enterica* Typhimurium in wild sparrows habituating near the farm [47]. As pointed out earlier, the ESBL-producing *E*. *coli* isolates from Swedish gulls resemble isolates from Swedish surface water used as drinking water sources. Wild gulls are waterfowls that feed and defecate in the water, and these settings could potentially serve as a dissemination route of ESBL-producing *E*. *coli* from wild birds back to humans. One can also not rule out other settings such as faeces-contaminated agricultural land fields and playgrounds for small children as transmission routes to humans.

## Conclusions

The genetic similarities to human isolates and high occurrence frequency of ESBL-producing *E*. *coli* in gulls suggest that ESBL-producing *E*. *coli* are a form of environmental pollution. The results of the study support the hypothesis that gulls can function as environmental reservoirs and as indicators of environmental contamination of ESBL genes. In addition, the results suggest that gulls could be used as indicators of what types of antibiotic resistance are circulating in a human population. Our findings highlight the need for efforts to minimize the risk of exposing wildlife to human waste and sewage to prevent further contamination and dissemination of antibiotic resistance.
